# Mutagenesis of tyrosine and di-leucine motifs in the HIV-1 envelope cytoplasmic domain results in a loss of Env-mediated fusion and infectivity

**DOI:** 10.1186/1742-4690-8-37

**Published:** 2011-05-14

**Authors:** Sushma J Bhakta, Liang Shang, Jessica L Prince, Daniel T Claiborne, Eric Hunter

**Affiliations:** 1 Emory Vaccine Center at the Yerkes National Primate Research Center and Department of Pathology and Laboratory Medicine, Emory University, Atlanta, Georgia 30329, USA

## Abstract

**Background:**

The gp41 component of the Human Immunodeficiency Virus (HIV) envelope glycoprotein (Env) contains a long cytoplasmic domain (CD) with multiple highly conserved tyrosine (Y) and dileucine (LL) motifs. Studies suggest that the motifs distal to major endocytosis motif (Y_712_HRL), located at residues 712-715 of Env, may contribute to Env functionality in the viral life cycle. In order to examine the biological contribution of these motifs in the biosynthesis, transport, and function of Env, we constructed two panels of mutants in which the conserved Y- and LL-motifs were sequentially substituted by alternative residues, either in the presence or absence of Y_712_. Additional mutants targeting individual motifs were then constructed.

**Results:**

All mutant Envs, when expressed in the absence of other viral proteins, maintained at least WT levels of Env surface staining by multiple antibodies. The Y_712 _mutation (Y712C) contributed to at least a 4-fold increase in surface expression for all mutants containing this change. Sequential mutagenesis of the Y- and LL-motifs resulted in a generally progressive decrease in Env fusogenicity. However, additive mutation of dileucine and tyrosine motifs beyond the tyrosine at residue 768 resulted in the most dramatic effects on Env incorporation into virions, viral infectivity, and virus fusion with target cells.

**Conclusions:**

From the studies reported here, we show that mutations of the Y- and LL-motifs, which effectively eliminate the amphipathic nature of the lytic peptide 2 (LLP2) domain or disrupt YW and LL motifs in a region spanning residues 795-803 (YWWNLLQYW), just C-terminal of LLP2, can dramatically interfere with biological functions of HIV-1 Env and abrogate virus replication. Because these mutant proteins are expressed at the cell surface, we conclude that tyrosine and di-leucine residues within the cytoplasmic domain of gp41 play critical roles in HIV-1 replication that are distinct from that of targeting the plasma membrane.

## Background

The envelope glycoprotein (Env) cytoplasmic domain (CD) is a key determinant in the replication of Human Immunodeficiency Virus type I (HIV-1) at two pivotal steps: (*i*) at the point of viral assembly, where Env must be incorporated into budding virions, and (*ii*) at the stage of viral entry into host target cells. The Env CD has been shown through both genetic and biochemical approaches to interact with domains of Gag during assembly [1-3], interact with cellular components during intracellular transport [4-7], modulate the fusogenicity of the Env complex both in the cell and within the virion [4,8,9], and regulate the cell surface expression of Env [10-13]. However, exactly which Env CD sequences mediate these phenotypically important roles remains to be elucidated.

Env, a type I transmembrane protein, is synthesized as the precursor protein, gp160, on ribosomes associated with the endoplasmic reticulum (ER) [14]. Upon oligomerization and correct folding of gp160 [14], the stable complex is then transported from the ER to the trans Golgi network, where Env is terminally glycosylated and then processed into gp120, the receptor-binding surface (SU) protein, and gp41, the trans-membrane (TM) component, by a furin-like protease [14]. In the mature form of Env, gp120 and gp41 are non-covalently linked. The mature Env complex, which facilitates viral entry into host cells [15,16], is then transported to and expressed on the cell surface, where either of two events may occur: Env is either incorporated into budding virions or it is rapidly internalized [10-13,17].

In the context of the mature virion, Env mediates virion attachment to the HIV-1 receptor, the CD4 molecule, and its chemokine co-receptor, CXCR4 or CCR5, and mediates fusion of the viral and cellular membranes [2,3,9,10,18], thereby facilitating entry of the virus into the host target cell. Viral infectivity depends on Env incorporation into budding virions and the subsequent entry into and infection of target cells.

Lentiviruses, such as HIV-1 and SIV, contain TM proteins with unusually long CD of ~150 amino acids (aa), in contrast to other retroviral TM CD, which are 20-40 aa long [14]. However, it remains unclear why these long cytoplasmic tails have been conserved. Truncation and elongation of the TM CD have been shown to alter the functionality of Env in the viral life cycle. Truncation studies reveal that the CD is dispensable for Env-mediated cell-cell fusion [3,19,20] and for SIV replication [21,22]. SIV growth in human cells selects for a spontaneously truncated Env, which broadens the host range of the virus [21,22]. However, the virus encoding the truncated Env reverts back to wild type (WT) upon inoculation into macaques [23]. This reversion back to WT suggests that while this region is dispensable *in vitro*, it plays an important role *in vivo*; and a number of structural elements within the CD may contribute to this *in vivo *function [24].

In HIV-1, truncation of the CD by as few as 20 amino acids significantly reduced viral replication in most cell types [2,19,20,25]. It is required in a cell-type dependent manner for incorporation of Env into virions and for generating a productive, transmissible infection in most of the T cell lines tested [3]. Cell-type dependence may be due to differences in expression and localization of host factors [26], suggesting that gp41 CD interactions with cellular proteins are important for efficient virus assembly. Similarly, it appears necessary for this region of Env to interact with the matrix (MA) domain of the Gag polyprotein precursor for incorporation of full-length proteins [1], which is supported by the fact that mutations in the CD, which block Env incorporation, can be rescued by amino acid changes in MA [3].

The HIV-1 gp41 CD contains several potential internalization and trafficking motifs, including four tyrosine motifs at 712_Yxxφ_, 768_Yxxφ_, 795_YW_, and 802_YW_, and six dileucine motifs at 774_LL_, 776_LI_, 784_LL_, 799_LL_, 814_LL_, and 855_LL_, that have been conserved in the majority of HIV-1 patient isolates. Both tyrosine-based (Yxxφ) and dileucine-based (LL) motifs can play individual or overlapping roles [27-29]. These overlapping roles are modulated by different requirements for proximity to trans-membrane domains and to the carboxy or amino terminus [30]. Residues near the motif itself can either strengthen or specialize the signal or the mediating interaction [30]. Thus while these motifs have been shown to facilitate endocytosis, basolateral targeting in polarized cells, and targeting to specialized compartments within the cells [30], dissecting out individual functions for each motif is complex.

The membrane proximal Yxxφ motif has been established as the major endocytosis signal for gp41 [11,12,31-33], which is suppressed in the presence of Pr55gag [17]. The Y712 motif has been shown to direct the basolateral targeting of Env and the polarized budding of HIV-1 [34,35] and to interact with the μ1 and μ2 chains of adaptin (AP) complexes [4,31,33]. Mutagenesis of this motif in both the HIV and SIV Env CD has consistently resulted in increased levels of cell surface expression [7,11,13,32,35], although in one study this resulted in decreased infectivity, entry, and poorly replicating virus, independent of Env incorporation into virions [36]. Further, a study in SIV demonstrated that abrogation of the membrane proximal Yxxφ motif through deletion of a highly conserved GY amino acid pair yielded replication competent virus that was highly attenuated *in vivo *[24].

The YW_802 _motif has been well studied and reported to interact with TIP47, implicated in linking the Env-Gag interaction [37], resulting in the retrograde transport of Env from the endosome to the Golgi [5]. Abrogation or deletion of YW_802 _also resulted in decreased Env incorporation, infectivity, and replication [3,38]. The C-terminal LL_855 _has also been shown to interact with AP-1 and to regulate the subcellular localization of Env [10], with varying reports regarding its role in the endocytosis of Env [10,39]. The Y_768_xxφ motif, in addition to LL_774_, LI_776_, and LL_784_, overlaps with the inhibitory sequence 2, *is2*, which has been described as inhibiting the surface expression of Env [40], although mutagenesis of Y_768 _alone did not result in a distinct phenotype or loss of AP-2 μ chain binding by Env [31]. Interestingly, this tyrosine motif resembles the "three-pin plug" tyrosine motif previously described for μ2 binding to the P-selectin protein [41], in that there is a similar upstream leucine residue (LxxY_768_xxL) that could also contribute to adaptin binding in the absence of the tyrosine.

A number of the conserved motifs also overlap with the amphipathic α-helical lentiviral lytic peptides LLP1 (aa 828-856) [42,43], LLP2 (aa 770-795) [44], and LLP3 (aa 789-815) [45]. This feature complicates mutational analyses since LLP1 and LLP2 have been reported to play a role in the fusion process [46]. Further complicating the biological role of the Env CD, is the novel finding that there is coupling of the fusion process with virion maturation [47] and that the Env CD also impedes the entry of immature virions into target cells through its interaction with the immature Gag core [46,48-50]. The complexity of these trafficking motifs, located within close proximity to each other and physically overlapping with other functional domains, exemplifies the difficulty in dissecting out the roles of the trafficking motifs conserved along the Env CD.

In order to better understand why HIV-1 has conserved tyrosine and di-leucine motifs within the unusually long CD of Env, we have employed a progressive mutagenesis strategy to sequentially mutate all of the conserved Y- and LL-based motifs in the gp41 CD, followed by more targeted mutagenesis of individual motifs. For each of these sequential mutants, we have determined surface expression, fusogenicity, incorporation, and the ability to facilitate entry and infection into target cells. Sequential mutagenesis generally resulted in progressive impairment of Env fusogenicity, Env incorporation, viral infectivity, and viral entry, despite efficient transport and expression of Env on the cell surface. The most dramatic phenotype was observed following mutation of Y_768_, and adjacent dileucine motifs within LLP2, which points to a critical role for the amphipathic nature of this region in modulating Env function. This was confirmed by targeted mutagenesis, which also identified a motif in LLP3 critical for virus entry and replication.

## Results

### Generation of Env mutants

**Figure 1:**
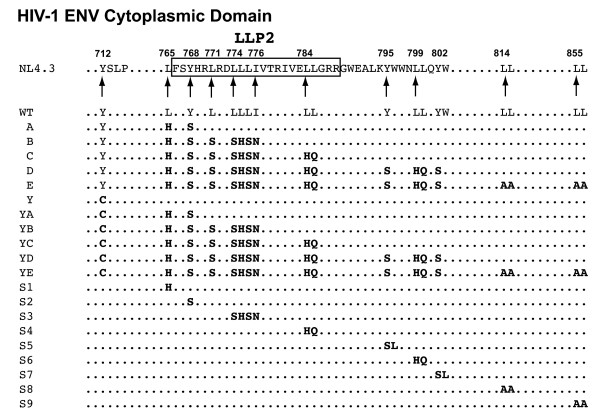
**HIV-1 Env cytoplasmic domain Y- and LL-motif mutagenesis strategy**. The key amino acids of the Y- and LL-motifs targeted for mutagenesis are listed under the corresponding location in the sequence of NL4-3 envelope cytoplasmic domain. WT residues are indicated by regular-faced type, and the residues in bold-faced type represent the mutations for each mutant. The glycoproteins are separated by those containing the WT Y_712 _motif and by those containing the Y712C mutation.

The unusually long CD of gp41 contains multiple Y- and LL-motifs. In order to define the functional role played by these motifs in the HIV-1 life cycle, a progressive mutagenesis strategy was employed in which the Y- and LL-based motifs were sequentially mutated along the Env CD. Several of these motifs overlap the Rev open reading frame, necessitating substitutions that maintain Rev function. The mutants were classified based on their location in the NL4-3 Env and are shown in Figure 1. Site-directed mutagenesis was employed to introduce the trafficking motif mutations into the *env *gene. A complex overlapping PCR strategy was then utilized to create progressive mutants in the CD. Introduction of the L765H/Y768S mutations into the *env *sequence generated mutant A. The subsequent addition of L771S/LLLI774SHSN to mutant A results in mutant B, the addition of LL784HQ to mutant B results in mutant C, the additional changes of Y795S/LL799HQ/Y802S to mutant C produce mutant D, and LL814AA/LL855AA was combined with mutant D to create mutant E. Introduction of the Y712C mutation to WT and the Env mutants A, B, C, D, and E resulted in the generation of the Y, YA, YB, YC, YD, and YE mutants. The role of individual motifs was then probed by an additional set of mutations (Figure 1). All Env CD mutants were cloned into the Env expression vectors pSRHS and pSRHS-EB, as well as the proviral vector pNL4.3.

### Envelope biosynthesis, processing, and stability

**Figure 2:**
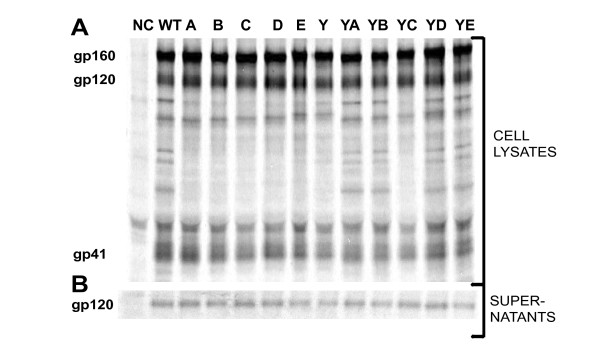
**Biosynthesis and processing of mutant glycoproteins**. COS-1 cells transiently transfected with the Env expression vector pSRHS were metabolically labeled in a pulse, followed by a 4 h chase and immunoprecipitated with anti-HIV-1 patient sera. The locations of the Env precursor and the components of the mature Env complex are indicated at the left of the gel. The pulse-chase cell lysates of glycoproteins expressed from the pSRHS vector (A) are shown in the gel at the top, and the corresponding gel for the amount of gp120 shed into the supernatant (B), is shown in the gel at the bottom.

In order to investigate the effects of this mutagenesis on the biosynthesis, processing, and stability of the glycoproteins, WT and mutant envelopes were expressed from the SV40-based pSRHS vector, which also expresses Rev and Tat. Env expression was under the control of the SV40 late promoter and polyadenylation signals were provided by the long terminal repeat (LTR) of the Mason-Pfizer monkey virus [19,21]. The WT and mutant glycoproteins were expressed in COS-1 cells, which have been shown to facilitate high expression of Env from pSRHS [19]. Two days after transfection, the Env proteins were metabolically labeled for 30 min with [^35^S] and further chased for 4 h in complete unlabeled media. Following lysis of the cells, the glycoproteins within the cell lysates and supernatants were immunoprecipitated with HIV-1 patient sera, resolved by SDS-PAGE, and visualized by autoradiography [19,21]. Sequential mutagenesis of the Y- and LL-based motifs in the CD mutants did not decrease the level of expression of gp160, or the processing of precursor to gp120 and gp41, indicating normal intracellular transport to the trans-Golgi network, as seen in a pulse-chase experiment in Figure 2A. Examination of the amount of gp120 shed into the supernatant also revealed that the mutagenesis of these motifs did not alter the stability of gp120, represented in Figure 2B. Similar results were seen in pulse-chase experiments conducted with the pSRHS-EB Env expression constructs (data not shown).

### Effects of sequential mutagenesis in the cytoplasmic domain of Env on cell-cell fusion

**Figure 3:**
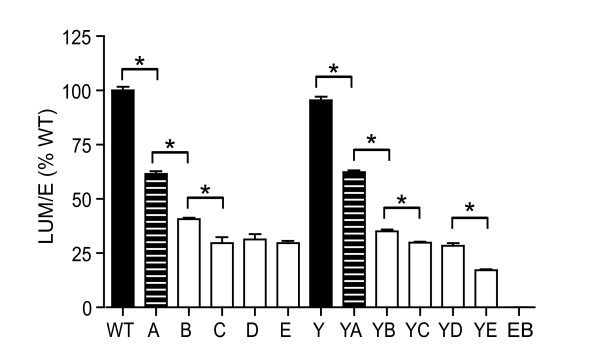
**Env-mediated cell-cell fusion**. COS-1 cells transiently transfected with the Env expression vector were cultured for 24 h. The COS-1 cells transiently expressing WT and mutant glycoproteins were co-cultured with TZM-bl indicator cells. Following a 24 h incubation, the co-culture of cells was lysed and measured for luciferase activity. P values were calculated by using Tukey's T-test and a value <0.001 are shown with an asterisk. The data represents results from three independent experiments conducted in triplicate. In these assays WT fusion yielded an average of 4.7 × 10^5 ^DLU and EBFP an average of 8.4 × 10^3 ^DLU. The error bars represent the standard deviation of the means.

Because the Env trafficking motif mutants maintained WT levels of biosynthesis, processing, and stability, we wanted to screen the glycoproteins for functionality. In order to measure Env-mediated cell-cell fusion, a luciferase-based fusion assay was utilized. The Env expression vector containing WT and mutant *env *genes, including both the *rev *and *tat *genes, was expressed in COS-1 cells. Two days after transfection, the transiently transfected COS-1 cells were co-cultured and mixed with TZM-bl indicator cells, which contain an HIV-2 LTR driven luciferase gene and express the HIV-1 receptor, CD4, and coreceptors CCR5 and CXCR4. Upon fusion of the cellular membranes of the Env expressing COS-1 cells and the target TZM-bl cells, Tat, which is also expressed from pSRHS-EB, activates the HIV-2 LTR and drives luciferase production [51]. A quantitative analysis of Envelope mediated cell-cell fusion was measured for each of the mutants by calculating their relative luciferase enzyme activity compared to WT. The relative luciferase activity for each of the mutants was averaged from three independent experiments performed in triplicate; these results are shown in Figure 3. The low background resulting from the EBFP control, expressed from the pEBFP-N1 construct lacking the *env*, *rev*, and *tat *genes, was subtracted from the experimental values to give a baseline for fusion activity.

In Figure 3, the Env mutants have been separated into two series, those containing the WT Y_712 _motif and those containing the Y712C mutation. Direct comparison of the two panels indicates that the Y712C mutation did not affect the fusogenicity of the Env mutants in the context of cell-cell fusion, with the Y mutant maintaining 96% fusion activity compared to WT. Mutagenesis of the first two pins (L765H and Y768S) in the three-pin motif LxxY_768_xxL, which binds to AP2, at the N-terminus of LLP2 resulted in 62% and 63% the fusogenicity of WT for mutants A and YA, respectively. Subsequent mutagenesis of the third pin (L771S) and the LL_774_LI_776 _motifs resulted in a significant decrease in fusion compared to WT, with B and YB reducing fusogenicity to 41% and 35% of WT respectively. Fusion activity decreased in the remaining mutants to approximately 30% that of WT, while mutant YE had a greater decrease at 17% of WT. Thus, sequential mutagenesis of the Y- and LL-based motifs within the long CD of HIV-1 Env resulted in a progressive decrease of Env mediated cell-cell fusion activity. These results show that mutation of the Y- and LL-based motifs contained within the Env CD can modulate fusion activity of the Env glycoprotein.

### Effects of mutagenesis in the cytoplasmic domain on Env cell surface expression

**Figure 4:**
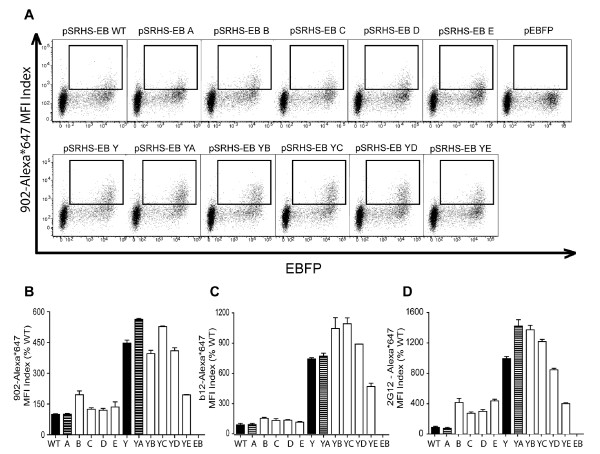
**Cell surface expression of envelope glycoproteins**. (A) COS-1 cells transiently transfected with each of the pSRHS-EB Env expression vectors were immunostained with Alexa*647-conjugated anti-gp120 mAb 902. The dot plot panels are separated into two series for analysis: (1) those containing the WT Y_712 _motif in the top row, and (2) those containing the Y712C motif in the bottom row. (B) The quantified surface expression levels of the Env glycoproteins are shown as the relative mean fluorescence intensity (MFI) Index (MFI x% of cells double positive for EBFP and Alexa*647). Additional cells were stained with Alexa*647-conjugated anti-gp120 mAb b12 (C) and anti-gp120 mAb 2G12 + Alexa*647-Goat anti-hu IgG (H+L) (D). The error bars represent the standard deviation of the means.

Because sequential mutagenesis of the trafficking motifs within the CD resulted in a progressive decrease in Env fusion activity, we wanted to establish whether this resulted from an altered transport to and expression on the cell surface. COS-1 cells expressing the WT and mutant envelopes were stained with each of three monoclonal antibodies (mAb): 902, which recognizes a linear epitope on the gp120 V3 loop [52,53], b12, which recognizes an epitope that overlaps the CD4 binding site [54,55], and 2G12, which recognizes a complex of carbohydrates on the surface of gp120 [56]. The first two were directly conjugated to AlexaFluor^®^647, while 2G12 was detected using Alexa647 labeled Goat anti-human IgG (H+L). Following immunostaining, the cells were subjected to flow cytometry analysis. EBFP expression from the Env expression vector served as the experimental transfection control. The results from the flow cytometry analysis are shown in Figure 4. Once again, the samples have been separated into two series: those containing the WT Y_712 _motif and those containing the Y712C mutation. The MFI Index value was calculated for each of the samples. The results indicate that all of the Env CD mutants maintained at least WT levels of surface expression, while introduction of the Y712C mutation into the CD resulted in an increase in glycoprotein cell surface expression, following immunostaining with all three antibodies. In Figure 4A, the flow cytometry dot plots of mAb 902 stained cells reveal a distinct shift in the staining pattern between the WT Y_712 _panels and the Y712C panels, with a greater proportion of the cells staining and with higher intensity in the latter, consistent with increased levels of surface expression. The corresponding MFI Index values are shown in Figure 4B. The MFI Index values for the WT Y_712 _panel of mutants were similar to WT Env levels with A at 101%, B at 195%, C at 125%, D at 120%, and E at 136% that of WT. By inserting the Y712C mutation into WT Env, the MFI Index value of the Y mutant increased to 447% of WT. This increase was reflected in the MFI Index values of the other mutants containing the Y712C, including YA at 563%, YB at 396%, YC at 563%, YD at 409%, and YE at 194%.

We confirmed the increased surface expression with the 2 additional monoclonal antibodies. The results for immunostaining with mAb b12 are shown in Figure 4C and those for mAb 2G12 in Figure 4D. The staining patterns for both antibodies are similar to that observed with mAb 902, with a majority of the Y_712_WT mutant-expressing cells exhibiting MFI indices similar to WT Env, although for 2G12 a 3-4-fold increase in surface staining was observed for mutants B-E. As with 902, a majority of the cells expressing the Y712C-containing mutants exhibited much higher levels of surface staining with b12 and 2G12, although the absolute increase differed (approximately 8 and 10-fold for Y, respectively). In each case, cells expressing the YE mutant showed the smallest increase in Env surface expression of the Y712C-containing mutants relative to WT.

### Env CD mutants exhibit a defect in virus entry and virus-cell fusion

Because the levels of surface expression of the Env CD mutants did not correspond to the observed defects in cell-cell fusion, we examined the Env mutants, in the context of pSG3*Δenv *pseudotyped virus, for their capacity to mediate virus entry and virus-cell fusion.

**Figure 5:**
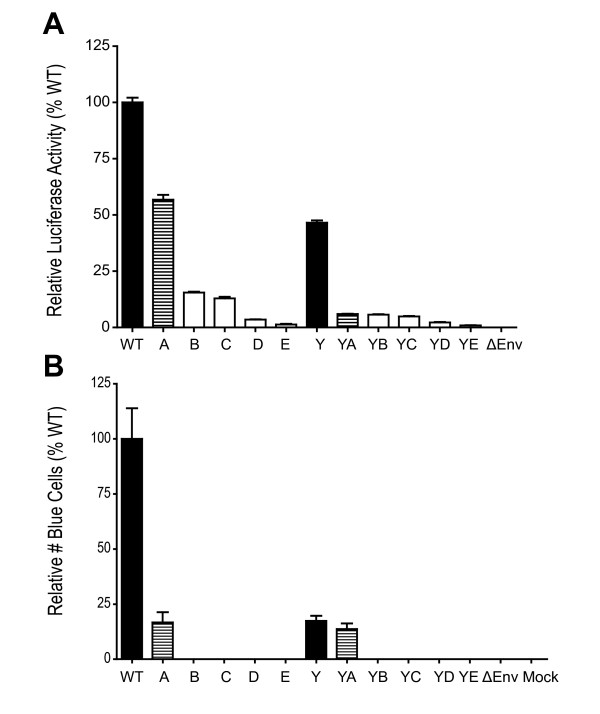
**Infectivity and entry of Env cytoplasmic domain mutants**. (A) Single round infectivity. Env-pseudotyped SG3ΔEnv viruses produced in 293T cells and p24-normalized, were used to infect TZM-bl indicator cells. After a 48 h incubation, the cell mixtures were lysed and luciferase activity was assayed. In this assay infection with virus pseudotyped with WT Env yielded 2.7 × 10^5 ^DLU and ∆Env virions an average of 1.65 × 10^4 ^DLU (B) Virus-cell fusion assay. Env CD mutants in NL4-3, pseudotyped with pCMV-BlaM-Vpr, were produced in 293T cells and subjected to gradient ultracentrifugation. Resuspended viral pellets were then normalized using p24 ELISA assays and used to infect TZM-bl indicator cells. The CCF2-AM fluorescent dye was loaded into the cells and incubated for 16 h at room temperature. The data represents results from three independent experiments conducted in triplicate. The error bars represent the standard deviation of the means. WT virus induced blue fluorescence in 5.48% of the 25,000 cells analyzed, a mock infected background of 0.46% blue cells was subtracted from all values.

A luciferase-based single round virus entry assay was conducted, utilizing the same target cell fusion system as described above. Equivalent amounts of pseudotyped virus (normalized for p24), produced in COS-1 cells, were used to infect TZM-bl indicator cells. The cells were measured for luciferase activity at 48 h post-infection. The SG3*Δenv *virus was used as the background control. The results indicate that the sequential mutagenesis of the Env CD trafficking motifs resulted in much more pronounced defective phenotypes in the context of pseudotyped virus as shown in Figure 5A. In contrast to the cell-cell fusion results, where the maximum decrease observed for mutant E was 70%, infectivity of virus pseudotyped by this Env was reduced 99%. Even mutant B, in which just the Y_768 _motif and two adjacent dileucine motifs are mutated, exhibited only 16% the virus entry activity of WT Env. While the Y712C substitution in mutant Y had little effect on cell-cell fusion, the infectivity of viruses pseudotyped with this Env was 47% that of WT, and the remaining Y712C-containing mutants were reduced in virus entry by more than 94% compared to WT.

In order to further define the defect in entry, we utilized the β-lactamase virus-cell fusion assay described previously [57-60]. For this assay, pNL4-3 proviral clones were co-transfected with a β-lactamase-Vpr fusion protein (BlaM-Vpr) expression vector, and the released virus was used to infect TZM-bl cells as described in Materials and Methods. The extent of virus-cell fusion, as assessed by intracellular β-lactamase activity, is shown in Figure 5B. The results of this assay were similar to those observed in the virus entry assay (Figure 5A), with only mutants A, Y and YA exhibiting low levels of β-lactamase activity, 14-17% that of WT.

### Glycoprotein incorporation into mutant virions is reduced

**Figure 6:**
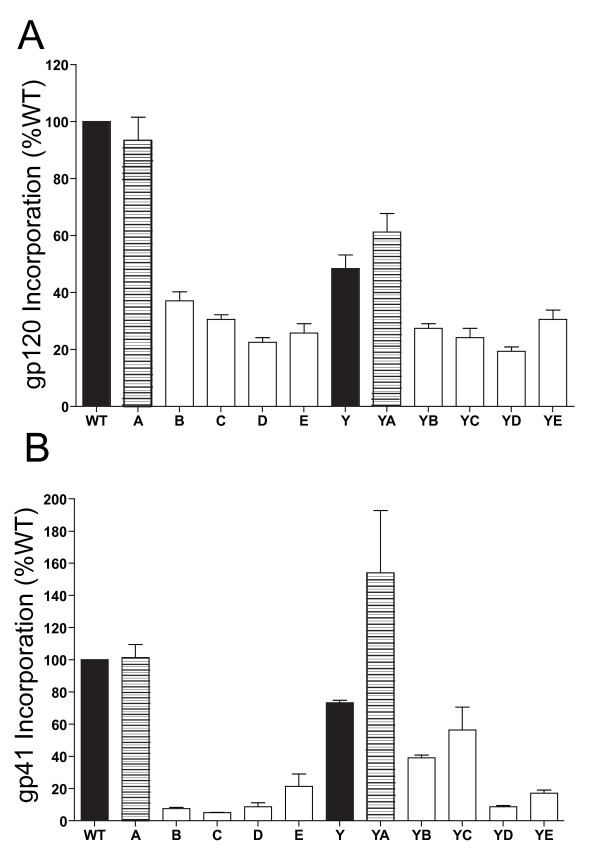
**Incorporation of Env glycoproteins into virions**. NL4-3 viruses containing the WT and mutant Env proteins were produced in 293T cells and pelleted through a 25% sucrose cushion. Resuspended viral pellets were then subjected to p24 ELISAs, gp120 ELISAs, and gp41 western blotting. Incorporation of Env into virions is calculated as the ratio of gp120/p24 (A) and gp41/p24 (B) and shown as the percentage of WT. The data represents results from three independent experiments conducted in triplicate. The error bars represent the standard deviation of the means.

To establish whether a defect in Env incorporation into virions contributed to the infectivity impairment of the Env CD mutants, we measured virus-associated gp120 and gp41 glycoprotein. The CD mutant viruses were recovered from provirus transfected 293T cells, pelleted through a 25% sucrose cushion, and then subjected to p24 and gp120 ELISAs and gp41 western blotting. The ratios of gp120/p24 and gp41/p24 were calculated for each virus to measure Env incorporation into virions, and are shown in Figure 6 as the percentage of WT. Mutant A incorporated near WT levels of both gp120 and gp41, but the levels of virus-associated glycoprotein rapidly decreased, with mutants B through E incorporating 24-38% the amount of gp120 and 5-22% gp41 compared to WT. The Y712C mutation reduced the level of gp120 and gp41 incorporation to 49% and 73% that of WT, respectively. Although, the level of virus-associated gp41 was increased in mutant YA (154% of WT), such mutations appeared to impair stability of Env complexes, since gp120 incorporation was only 73% of WT. The addition of the Y712C mutation facilitated gp41 incorporation in mutant YB and YC, compared to their non-Y712C counterparts, while mutants YD and YE showed similar gp41 levels to their non-Y712 counterparts.

### Effects of individual tyrosine and di-leucine mutants in the Env cytoplasmic domain

Because mutations beyond B or YB exhibited only limited additional phenotypic defectiveness, we performed additional mutagenesis of individual motifs to investigate whether they could significantly influence the functions of HIV-1 Env. As shown in Figure 1, nine additional single-motif mutants were constructed. Mutations in S1 (L765H), S2 (Y768S), S3 (LLLI774SHSN), and S4 (LL784HQ) are located in the N-terminus (S1, S2), middle (S3) and C-terminus (S4) of the LLP2 motif. Mutants S5 (YW795SL), S6 (LL799HQ), S7 (YW802SL), S8 (LL814AA), and S9 (LL855AA) target the other Y- and LL-motifs downstream of the "three-pin" motif. Cell-cell fusion and single-round infection mediated by these Env mutants were measured and compared to WT using the same methods as described for the progressive mutants.

**Figure 7:**
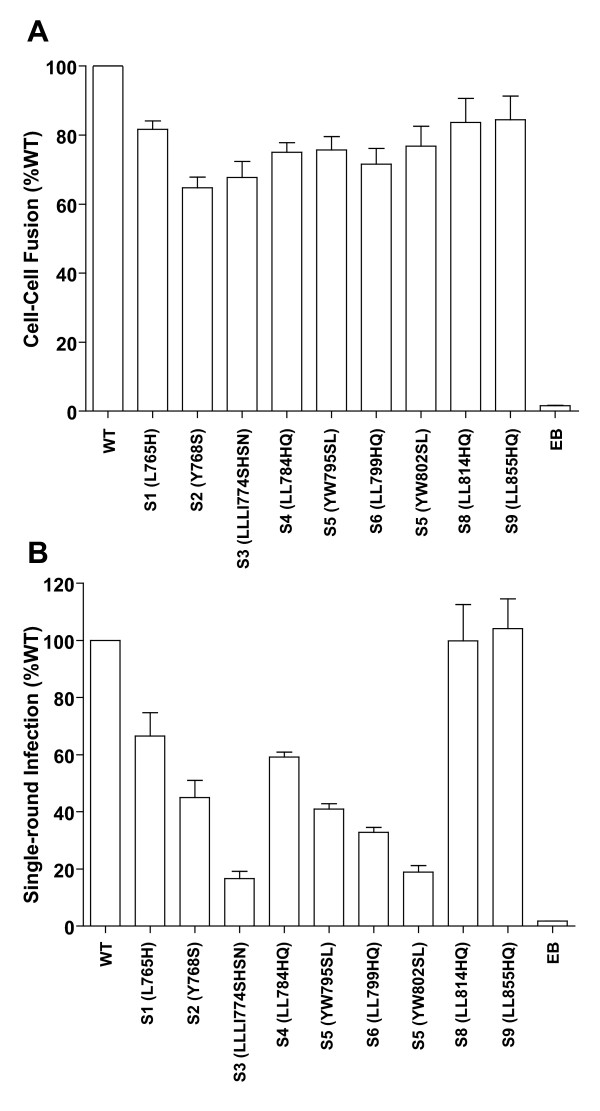
**Single-motif mutants in the Env CD**. (A) Cell-cell fusion mediated by Env carrying the single-motif mutants. (B) Single-round infection of viruses SG3Δ*env*, which were pseudotyped with single-motif Env mutants, in TZM-bl indicator cells. The error bars represent the standard deviation of the means.

As shown in Figure 7A, each single motif is required for WT level of Env-mediated cell-cell fusion; however, Env fusogenicity is not dominated by a particular single motif. Most of the single-motif mutants retained 75% to 85% of WT cell-cell fusion. The integrity of the LLP2 motif appeared most important for Env fusogenicity since mutants S2 (Y768S) and S3 (LLLI774SHSN) retained the lowest level of cell-cell fusogenicity, 64.7% and 67.8% of WT, respectively.

Consistent with its function in Env-mediated cell-cell fusion, the LLP2 motif is also very important for virus entry (Figure 7B). The loss of hydrophobicity in mutants S1 (L765H), S3 (LLLI774SHSN), and S4 (LL784HQ) significantly reduced the single-round viral infection to 66.5%, 16.6%, and 59.2% of WT. Meanwhile, the mutant S2 (Y768S) exhibited only 45% WT efficiency of virus entry. Other motifs, including YW795, LL799, and YW802, appeared critical for virus entry as well. Mutants S5 (YW795SL), S6 (LL799HQ), and S7 (YW802SL) retained only 19%-32.9% WT efficiency in the single-round infection assay. Interestingly, the motifs LL814 and LL855 did not appear to be necessary for virus entry, even though they reduced Env-mediated cell-cell fusion to a small extent. These data indicate that a majority of the Y- and LL-motifs in the Env CD contribute to decreases in viral infectivity as mutations accumulate.

### Effects of individual motif mutations on virus replication in T cells

**Figure 8:**
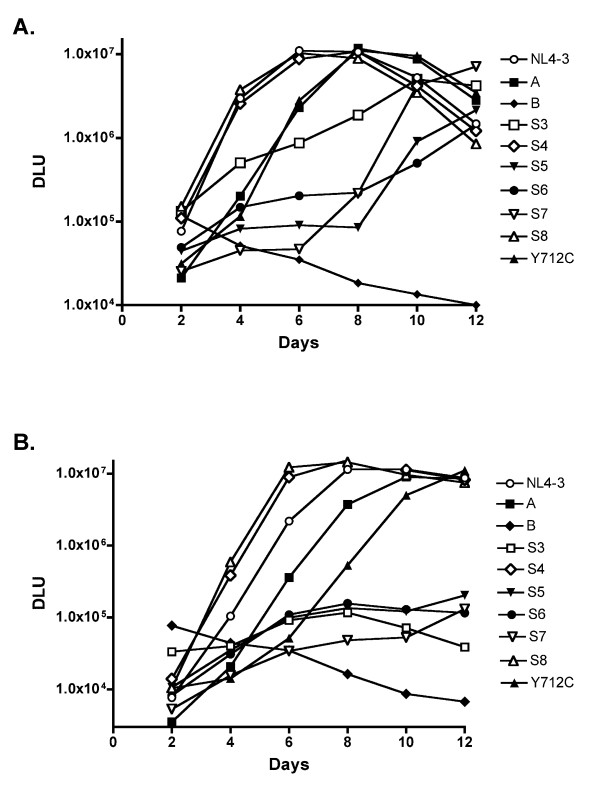
**Replication in CEM (A) and H9 (B) cells**. Viruses were generated by transfecting 293T cells with proviral DNAs. T cell lines were infected with an M.O.I. of 0.05. The supernatants were collected at day 2, 4, 6, 8, 10, and 12, then subjected to a reverse transcriptase assay to quantitate the amount of virions released at each time point.

In order to examine the influence of the Env CD mutants on virus replication, we measured the replication kinetics of these mutants over a period of 12 days in both the H9 and CEM T cell lines by a reverse transcriptase assay. NL4-3 proviral constructs were transfected into 293T cells, supernatant virus was titered on TZM-bl cells, then CEM or H9 cells were infected with an equal MOI (0.05). As shown in Figure 8A, in CEM cells, single-motif mutants, S4 (LL784HQ) and S8 (LL814AA) showed similar replication capacities in H9 cells, with an initial replication rate comparable to WT. Mutants Y and A, as might be predicted from single round infections, demonstrated delayed replication kinetics, with peak RT values equivalent to, but 2 days after, WT. The additional mutation of the hydrophobic core of LLP2 in mutant B completely abrogated viral replication, with RT values dropping progressively over the course of the experiment, indicative of the infection of cells by the initial inoculum but then loss of RT production because the virus is unable to assemble infectious virus in T cells. The fact that mutant S3 (LLLI774SHSN) exhibits a significant but incomplete replication defect in CEM cells suggests that combining these mutations with mutant A, as in mutant B, is highly detrimental to the virus. Three mutants S5 (YW795SL), S6 (LL799HQ), and S7 (YW802SL) demonstrated a 6-8 day-delay before virus replication accelerated - and for S5 and S6 the peak of virus remained approximately 10-fold below that of WT.

Similar patterns of replication were observed in H9 cells (Figure 8B), except that mutants S3, S5, S6 and S7 exhibited much greater defects in replication, with peak RT values approximately 100-fold less than that of WT. Thus, in these cells, simply mutating the hydrophobic core of LLP2 (mutant S3) or any of the individual tyrosine or di-leucine motifs in LLP3 effectively abrogates virus infectivity.

## Discussion

The objective of this study was to investigate the role of the highly conserved Y- and LL-based motifs within the gp41 cytoplasmic domain (CD) in the HIV-1 life cycle. To this end, we have employed a progressive mutagenesis strategy, in which all of these motifs were sequentially mutated throughout the CD, and have followed this up with mutagenesis of individual motifs to probe additional function. Previous studies have attempted to study the role of the CD in the context of chimeric proteins [4,10,11,39,40], while others have truncated the CD in order to determine the affects on Env functionality [19,61-64]. However, while such an approach allows removal of all currently known trafficking motifs in the CD, there appears to be a functional dependence between the gp41 CD and its ectodomain, as well as a conformational dependence of gp120 on the Env CD [65]. This makes studying Env in the context of the full-length CD even more crucial. Truncation of the CD results in an increased susceptibility to neutralization by antibodies, likely due to a more open trimer conformation [55,66], and an increase in viral entry by non-replicating immature virions [47,50]. Similar studies also demonstrated that production of fully infectious virus requires the long CD [26].

Env glycoprotein biosynthesis, processing, stability, and transport to the Golgi (based on cleavage of gp160 to gp120 and gp41) were unaffected by the mutation of trafficking motifs. These motifs also appear, for the most part, to be dispensable for transport of Env to the cell surface. The Y_712 _motif, however, appears to be important for regulating the cell surface expression of the HIV-1 Env, as evidenced by a minimum 4-fold increase in surface expression of the Y (Y712C) mutant. Because the b12 mAb binds to an epitope that overlaps with the CD4-binding site on gp120, and because we were concerned with the structural dependence of gp120 on the gp41 CD, we performed surface immunostaining with three monoclonal antibodies, including mAb 902 and mAb 2G12, which bind a linear protein epitope and a complex carbohydrate epitope, respectively. All three mAb showed an increase in surface expression of the Y-mutants compared to the WT Y_712 _mutant panel, and a slight decrease in YE compared to the rest of the Y-mutants. All of the mutants maintained at least WT levels of surface expression in COS-1 cells, while all of the Y-mutants exhibited an increase in surface expression. This is consistent with previous studies of the membrane proximal Yxxφ motif in Env of both HIV and SIV [7,10,11,13,32,35].

A consistently lower level of surface staining relative to the other Y-mutants was observed for the YE mutant, even though this still exceeded that of WT Env for each mAb. In contrast, this was not observed for the E mutant, which exhibited surface staining levels equivalent to the B, C, and D mutants. Because YE lacks any of the conserved Y- and LL-based trafficking motifs, and so is unlikely to be more efficiently endocytosed, the reduced surface staining is most easily explained by less efficient transport of this mutant to the PM, perhaps because in the absence of Y_712 _necessary adaptin interactions are impaired.

Despite an increase in surface expression in the Y712C-containing mutants, there was a progressive decrease in Env fusogenicity from WT through C, after which Env fusogenicity stabilized (summarized in Table 1). Similar results were observed with the Y-mutants, although the mutant YE again was the most defective. Thus, changes in these tyrosine and dileucine motifs within the cytoplasmic domain are capable of inducing phenotypic effects on an event that is commonly associated with the ectodomain of Env (receptor and co-receptor binding, 6-helix bundle formation). The motifs mutated in A, B, and C are also of interest because they overlap with the LLP2 motif (aa 765-788; see Figure 1) in the NL4-3 gp41 CD, which has been proposed to play a role in fusion [46,67]. Indeed, Lu et al., [46] showed that at sub-optimal temperatures (31.5°C), antibodies to this region could bind to the interface of fusing cells and inhibit fusion. They proposed that, following formation of the gp41 HR1/HR2 6-helix bundle, the LLP2 peptide region is transiently exposed and modulates fusion by interacting with this helical complex. Consistent with this model, it is of interest that the reduction in fusion we observed for the CD mutants described here is maximal in mutant C (or YC), in which 7/9 hydrophobic residues within LLP2 are mutated and where the amphipathic nature of this region has been completely abrogated.

**Table 1:** Summary of the Functional Properties of HIV-1 Env Mutants

Env Mutants	Cell-Cell Fusion (%)	Surface Expression (mAb 902) (%)	Infectivity (pseudotyped) (%)	Virus-cell Fusion (%)	gp41 Incorporation (%)
WT	100	100	100	100	100
A	62	101	57	17	101
B	41	195	16	0	8
C	30	125	13	0	5
D	31	120	4	17	9
E	30	136	1	0	22
Y	96	447	47	0	73
YA	62	563	6	14	154
YB	35	396	6	0	39
YC	30	563	5	0	56
YD	29	495	2	0	9
YE	17	194	1	0	17

The effect of the CD mutations on viral infectivity in TZM-bl cells was much more pronounced than on cell-cell fusion (summarized in Table 1). In assays of Env pseudotyped virus, significantly reduced levels of infectivity were observed for all of the mutants. The A and Y mutants retained approximately 50% infectivity in pseudotyped virus assays, but the remaining mutants exhibited less than 20% (16-1%) that of WT. The defective stage in virus entry appeared to be at the level of virus-cell fusion, since the results of BLAM assays closely paralleled the infectivity results observed, in that only A, Y, and YA exhibited any virus-cell fusion and only at a level of approximately 20% that of WT.

It seems likely that the defects in virus infectivity represent the sum of defects in Env fusion and reduced levels of Env incorporation into virions (Table 1). Env incorporation decreased as more motifs were mutated, with the greatest drop being observed between mutants A and B (and in parallel between YA and YB). This is again consistent with a role for the hydrophobic residues within LLP2 region of the CD, since in mutants B and YB all of the hydrophobic residues in the N-terminal half of this region have been mutated to polar residues.

The Y mutant virions also showed reduced levels of Env incorporation, similar to that described in previous studies [7,36,38]. This result seems paradoxical to our observation of increased levels of Env at the cell surface, which is where virus buds [68]. The basis for reduced levels of Env incorporation are at present unclear, although it may reflect altered intracellular trafficking of Env and an inability of Env and Gag to be directed to a common site for assembly. Env clearly has the capacity to redirect where virus assembly occurs in the cell. In polarized epithelial cells, Env directs budding to the basolateral membrane and in CD4 T cells to a single pole of the cell [34,35,69,70]. Mutation of the major endocytosis motif at Y712 has been shown to disrupt polarized budding in both systems [34,35,70]. The loss of additional tyrosine and di-leucine motifs in mutants B-E (and YB-YE) could alter potential interactions of LLP2 with LLP1 and the membrane [71], which might further reduce the potential for co-localization of Env and Gag, and explain the observed reduction in incorporation.

Studies on single-motif mutants unraveled important information hidden in the approach of cumulative mutagenesis. An analysis of Env-mediated cell-cell fusion showed that a majority of the Y- and LL-motifs in the CD, when mutated individually, had only a limited effect on this function. From the observed decrease in cell-cell fusion with mutants A and B, as well as YA and YB, it appears that combinations of these changes can result in a more pronounced phenotype. This suggests that the single motifs may together contribute to form a functional structure, which is critical to HIV-1 Env-mediated cell-cell fusion.

In contrast to cell-cell fusion, virus replication is clearly impacted by some dominant single motifs. Three of these motifs (Y768XXL, L774LLI, and L784L) maintain the hydrophobicity of the Env CD, specifically in the LLP2 region, which is critically important for replication in T-cells. Whether mutation of this region prevents a translocation of LLP2 across the membrane as suggested by Lu et al. [46], or whether it prevents the region from mediating close membrane proximity of the Env CD, or interactions with other regions of the CD is not clear. Additional studies to define the exact mechanism of LLP2 function during virus replication are clearly warranted.

A second region of clustered tyrosine and di-leucine motifs is just C-terminal of the LLP2 region in LLP3. Mutation of either YW motif (mutant S5 and S7) or the LL motif (mutant S6) in this nine amino acid region (YWWNLLQYW) had a very significant impact on HIV-1 replication in T cells. This is consistent with previous results from Murakami and Freed (ref 2), who constructed overlapping deletions in this region, which also abrogated infectivity of HIV-1. Additional studies have focused on the YW802 motif, which has been postulated to interact with the cellular trafficking protein TIP47 in retrograde transport of Env from the endosome to the Golgi [5]. Mutation of the motif in Env or silencing of TIP47 expression resulted in reduced Env incorporation and virus infectivity [5,37]. In the studies presented here, although we did not observe any additional reduction in Env incorporation following mutagenesis of YW802 in mutant D, mutant S7 did exhibit delayed replication kinetics in CEM cells and very limited replication in H9 cells compared to WT, consistent with these previous studies. Nevertheless, it is clear that this entire nine amino acid region, not just YW802 is important for HIV-1 replication. Interestingly, only a limited effect of the S5-S7 mutations was observed in single round infections, suggesting that the constraints on Env-Gag interactions in 293T cells, where virus for these assays are produced, are less stringent than that in T-cells.

Overall, these studies point to two regions in the HIV-1 Env CD in which tyrosine and di-leucine motifs play critical roles in the biological function of the protein that are distinct from that of intracellular targeting of Env from the endoplasmic reticulum to the plasma membrane, thereby raising the possibility of therapeutically targeting these sequences in the future.

## Conclusions

From the studies reported here, we show that sequential mutagenesis of the Y- and LL-based motifs located within the CD of HIV-1 gp41 had a profound effect on Env function and demonstrates a critical role for hydrophobic residues in this region of the CD. This was evident in decreased Env-mediated cell-cell fusion, Env incorporation into virions, viral entry into target cells, and virus replication in T-cells. Env transport to the plasma membrane occurred in the absence of all of the conserved Y and LL motifs in the CD, arguing against a critical role for them in outward transport of the protein. Plasma membrane location alone was clearly not sufficient for efficient assembly of Env into virions, since a majority of the mutants exhibited reduced levels of Env incorporation and this, coupled with decreased fusogenicity of Env, resulted in them being non-infectious. The greatest phenotypic effects were linked to multiple changes in the LLP2 region of the CD and a region just C-terminal to this domain, which includes two YW motifs and a dileucine motif. Additional experiments will be required to determine whether the phenotypic defect resulting from changes in LLP2 reflects a distinct role for this region in late stages of Env-induced cell fusion, an alteration in CD-membrane interactions, or changes in protein-protein interactions within or between gp41 monomers necessary for the fusion process. Similarly, further analysis of the down-stream region, which has been implicated in binding the cellular protein TIP47, is clearly warranted. Overall, these studies highlight two regions in the HIV-1 Env CD in which tyrosine and di-leucine motifs play critical roles in the biological function of the protein and where changes in the context of the full-length domain have dramatic effects on virus replicative capacity.

## Methods

### Cell lines and culture

COS-1 and 293T cells were obtained from the American Type Culture Collection (Manassas, Va.), and TZM-bl were obtained through the NIH AIDS Research and Reference Reagent Program, Division of AIDS, NIAID, NIH: TZM-bl from Dr. John C. Kappes, Dr. Xiaoyun Wu and Tranzyme Inc. [51,72]. Cells were maintained in complete Dulbecco's modified Eagle's medium (DMEM) supplemented with 10% fetal bovine serum (HyClone Laboratories, Logan, UT), and 100 U/ml penicillin G sodium, and 100 μg/ml streptomycin sulfate (Gibco BRL, Rockville, MD), at 37°C and 5% CO_2_. All transfections were performed using the Fugene 6 (Roche, Indianapolis, IN) protocol at ~70% confluency of cells. All infections were conducted in DMEM containing 1% FBS and 80 μg/ml DEAE-dextran.

### Antibodies

The following reagents were obtained through the NIH AIDS Research and Reference Reagent Program, Division of AIDS, NIAID, NIH: HIV-1 gp120 Monoclonal Antibody (IgG1 b12) from Dr. Dennis Burton and Carlos Barbas [55,73-75], Hybridoma 902 (anti-gp120) from Dr. Bruce Chesebro [52,76], HIV-1 gp120 Monoclonal Antibody (2G12) from Dr. Herman Katinger [56,77], HIV-1 p24 Monoclonal Antibody (183-H12-5C) from Dr. Bruce Chesebro and Kathy Wehrly [53,78,79], and HIV-IG from NABI and NHLBI. The HIV-1 patient sera were obtained through the Emory CFAR Clinical Core. The horseradish peroxidase conjugated goat anti-human (heavy and light chains) mAb and the sheep anti-HIV-1 gp120 Polyclonal Antibody were purchased from Pierce (Rockford, IL) and Cliniqa Corp (San Marcos, CA), respectively. The Anti-HIV-1 gp120 D7324 mAb was purchased from Aalto Bio Reagents Ltd (Dublin, IE). AlexaFluor^®^647 Goat anti-human IgG (Heavy and Long Chain) was purchased from Invitrogen (Carlsbad, CA).

### Glycoprotein and proviral expression constructs

The HIV-1 Env CD trafficking motif mutants were generated by employing either a Quickchange PCR mutagenesis strategy (Stratagene, La Jolla, CA) or a multi-step overlapping PCR mutgenesis strategy using Expand High Fidelity PCR System (Roche, Indianapolis, IN). The resulting Env CD clones are referred to as follows: WT, Y, A, B, C, D, E, YA, YB, YC, YD, and YE. The second open reading frame (ORF) of *tat*, which overlaps with the gp41 CD between the motifs at 712 and 768, is unaffected by the substitutions made in these Env constructs. Because *rev *contains a second ORF that overlaps with seven of the ten trafficking motifs within the Env CD, the mutagenesis strategy employed focused on maintaining the integrity of *rev *while mutating out the Y and LL motifs within Env (Figure 1). The following primers were used for mutagenesis:

Y712CFP - 5'GGCAGGGATGTTCACCATTATCG3',

Y712CRP - 5'CGATAATGGTGAACATCCCTGCCTAACTC3',

LY768HSFP - 5'GCCTGTGCCACTTCAGCTCCCACCGC3',

LY768HSRP - 5'GCGGTGGGAGCTGAAGTGGCACAGGC3',

L771/LLLI774SHSSFP - 5'GCTCCCACCGCTCGAAAGACTCACACTCGAATGTAACGAGG3',

L771/LLLI774SHSSRP - 5'CCTCGTTACATTCGAGTGTGAGTCTTTCGAGCGGTGGGAGC3',

LL784HQFP - 5'CGAGGATTGTGGAACTTCTGGGACGCAGGGGG3',

LL784HQRP - 5'CCCCCTGCGTCCCAGAAGTTCCACAATCCTCG3',

Y795S/LL799HQ/Y802SFP - 5'GGAAGCCCTCAAACTTGGTGGAATCACCAACAGTCTTGGAGTCAGG3',

Y795S/LL799HQ/Y802SRP - 5'CCTGACTCCAAGACTGTTGGTGATTCCACCAAGATTTGAGGGCTTCC3',

LL814AAFP - 5'GC TGTTAACGCGGCCAATGCCAATGCCACAGC3',

LL814AARP - 5'GGCATTGGCCGCGT TAACAGCACTATTC3',

LL855AAFP - 5'GGGCTTGGAAAGGATTGCGGCATAAGATGGG3',

LL855ARP - 5'CCCATCTTATGCCGCAATCCTTTCCAAGCCC3'

All Env CD mutants were created in or from pSPEX-NL, a pSP-based vector (Promega, Madison, WI) containing the *Eco*RI-*Xho*I sequences of HIV-1 NL4-3, including the full-length cytoplasmic tail. Subsequent to verification in pSPEX, the mutant PCR fragments were subcloned at the unique sites *Nhe*I to *Xho*I from the pSPEX shuttle vector to the mammalian expression vector pSRH, a simian virus 40 late-promoter-based expression vector containing the Mason-Pfizer Monkey Virus constitutive transport element, to create the pSRHS construct, which expresses a full-length Env from NL4-3. The HIV-1 Env expression vector also encodes the *tat *and *rev *genes from NL4.3 [8].

To measure the surface expression of the mutant Env glycoproteins, an EBFP expression cassette was cloned into the pSRHS vectors at the unique restriction sites *NheI *and *BlpI *to create the pSRHS-EB vectors. The EBFP cassette was excised from the previously described vector [80]. For use in single-round infectivity and Env incorporation assays, the mutant Envs were also cloned into the proviral vector pNL4-3 via the unique sites *Nhe*I and *Blp*I. All mutations were confirmed by DNA sequencing and by using primers that flank the Env CD.

### Glycoprotein expression and immunoprecipitation

Env trafficking motif mutants in pSRHS expression vectors were transfected into COS-1 cells (2.5 × 10^5^) seeded in 6-well plates. To verify protein expression, processing, and stability, the transfected cells were metabolically labeled 36-48 hours posttransfection. The transfected cells were starved for 15 min in methionine-free and cysteine-free DMEM (Gibco-BRL, Rockville, MD) and pulse-labeled for 30 min in the same medium supplemented with [^35^S]Methionine and [^35^S]Cysteine (125 μCi/well) protein-labeling mix (Perkin-Elmer NEN, Boston, MA). The labeled cells were then chased for 4 h in unlabeled complete DMEM. The chase supernatants were removed and filtered through a 0.45 μm membrane to remove cellular debris. Cell lysates were prepared on ice by addition of 0.5 ml ice-cold lysis buffer (1% Triton X-100, 50 mM NaCl in 25 mM Tris-HCl [pH 8.0]), and nuclei were removed from lysates by centrifugation at 13,200 rpm for 10 min at 4°C in a microcentrifuge (Beckman, Palo Alto, CA). HIV-1 Env proteins were immunoprecipitated from cell lysates and supernatants by incubating at 4°C with HIV-1 patient sera. Immunoprecipitated proteins were then precipitated with formalin-fixed *Staphylococcus aureus *(protein A) and washed three times in lysis buffer containing 0.1% sodium dodecyl sulfate (SDS). The labeled proteins were resolved by 10% SDS-PAGE, visualized by autoradiography, and quantified using a Cyclone phosphorimaging system (Packard, Meridian, CT) as previously described [81].

### Cell-cell fusion assay

COS-1 cells (2.5 × 10^5^) were seeded in 6-well plates, transfected with the pSRHS-EB Env expression vectors at ~70% confluency, resuspended by trypsinization, and co-cultured with TZM-bl cells at a ratio of 1:5. The co-cultures of cells were incubated for 24 h and then lysed in the luciferase reporter buffer (Promega, Madison, WI). The cells were twice subjected to freezing for 1 hour and then thawing for 20 min, followed by centrifugation (Beckman, Palo Alto, CA) of the lysates at 13,200 rpm for 10 min to remove any cellular debris. Each cell lysate (40 μl) was added to a well in a 96-well plate, and then combined with 100 μl of the luciferase substrate (Promega, Madison, WI). Light emission was then measured using a Synergy multi-detector microplate reader (Biotek, Vinooski, VT) as previously described [59].

### Cell surface expression of Env glycoprotein

Surface expression of WT and mutant Env glycoproteins was measured using Flow cytometry in both a primary and secondary antibody (Ab) detection system. Env surface expression was measured by the human anti-gp120 mAb b12 and the mouse anti-gp120 mAb 902 each conjugated to AlexaFluor^®^647 (Invitrogen, Carlsbad, CA) in a primary detection system. The human 2G12 mAb was used in conjunction with the AlexaFluor^®^647 Goat anti-human IgG (H+L) to measure Env surface expression in a secondary Ab detection system. The Env proteins were expressed from the pSRHS-EB vector. EBFP expression served as a positive transfection control for these experiments. COS-1 cells were transiently transfected with pSRHS-EB and cultured for 36-48 h. Cells were then resuspended by trypsinization, washed three times, and stained for 1 h at RT with 5 μg/ml of the primary Ab. Cells stained with b12-Alexa^®^647 or 902-AlexaFluor^®^647 were washed three times prior to flow cytometry analysis. Cells stained with 2G12 were washed three times and then stained with the secondary Ab, AlexaFluor^®^647 Goat anti-human IgG (H+L), at 2 μg/ml for 1 h at RT. Double-stained cells were washed three times. Env surface expression was measured by flow cytometry analysis utilizing the LSRII system and the FACSDiva software (version 6.1), and analyzed using FlowJo software (version 8.8.4). Samples for each mutant were stained in triplicate, and a total of 50,000 events were accumulated for each sample. For each of these experiments, the mean fluorescence intensity (MFI) was calculated and multiplied by the percent of the cell population positive for both EBFP and R (660/20), to produce the MFI Index [59].

### Single-round infection

Single round infectivity was measured in a luciferase-based virus-cell fusion assay. COS-1 cells were seeded at a density of 2.5 × 10^5 ^in 6-well plates and co-transfected with the pSRHS expression vector and the pSG3Δ*env *proviral vector. The pSG3Δ*env *proviral vector was used as a negative control. At 72 h posttransfection, viral supernatants were clarified by centrifugation at 3,000 rpm for 20 min at 4°C to remove cellular debris. TZM-bl indicator cells (1 × 10^5^) seeded in 12-well plates were then infected with equivalent amounts of virus (5 ng p24), which were normalized by p24 enzyme-linked immunosorbent assay (ELISA) [59,82]. Complete DMEM was added after a 2 h incubation at 37°C, and luciferase activity was measured 48 h post infection as described above.

### Multi-round replication of Env mutants on CEM (GXR25) and H9 cells

Replicative capacity was assessed by infecting H9 and CEM-GXR25 cells (kindly provided by Dr. Mark Brockman, Simon Frazier University). Virus stocks for replication assays were generated using the following method: 1 μg of proviral DNA was transfected into a 70% confluent monolayer of 293T cells using the Fugene HD transfection reagent according to the manufacturer's protocol. Supernatants were collected 48 hrs post-transfection, clarified by low speed centrifugation, and stored at -80°C. The titer of each virus stock was determined by infecting TZM-bl cells with 3-fold serial dilutions of virus. Infectious units per μl (IU/μl) were determined for each virus stock by counting blue foci in the infected monolayers 48 hrs post-infection.

The day before replication assays, cells were split to 3 × 10^5 ^cells/mL. 5 × 10^5 ^cells were infected at an MOI of 0.05 calculated based on the IU/μl determined by infection of TZM-bl cells. Cells were infected in a total volume of 200 uL in a 96 well plate, using complete RPMI and 5 μg/mL of Polybrene. Cells and virus were incubated at 37°C for three hours, subsequently washed 4x to remove excess virus, and plated in 24-well plates at a total volume of 1 mL. Culture supernatants were collected and stored at -80°C on days 2, 4, 6, 8, 10 and 12 for viral quantification using a radiolabled reverse transcriptase assay. Cells were split every two days and replaced with fresh complete RPMI in order to maintain cell confluency. GXR25 cells were split 1:2 while H9 cells were spit 2:3

### Reverse transcriptase assay

Aliquots of culture supernatants from infected cells were added to an RT-PCR master mix and incubated at 37 degrees for 2 hours; then the RT-PCR product was blotted onto DE-81 paper, and allowed to dry. Blots were washed 5 times with 1 × SSC (0.15 M NaCl, 0.015 M sodium citrate, pH 7.0) and 3× with 90% ethanol, allowed to dry, and exposed to a phophsoscreen overnight. Counts were read using a Cyclone PhosphorImager.

### Virus-cell fusion assay

A virion-based fusion assay was performed as previously described by Cavrois [57-59]. BlaM-Vpr incorporated NL4.3 viruses were produced by transient co-transfection of the proviral plasmid pNL4.3, the pCMV-BlaM-Vpr vector (kindly provided by W. Greene, UCSF), and the pAdvantage vector (Promega, Madison, WI) by employing calcium phosphate precipitation of the DNA. BlaM-Vpr incorporated viruses containing WT and mutant Env glycoproteins were collected 48 h post-transfection and filtered through a 0.45-μm membrane. Viral supernatants were then loaded onto a 25% sucrose cushion (in PBS [pH 7.2]) and centrifuged at 100,000 × g for 2.5 h at 4°C as described above. The supernatant and sucrose layers were then removed and the resulting viral pellets were resuspended in serum-free DMEM. The virus titers were normalized by p24 ELISAs, and equivalent amounts of virus (200 ng p24) were then added to TZM-bl cells (3 × 10^5^), which were cultured in CO_2_-independent medium (Gibco-BRL, Rockville, MD) supplemented with 1% fetal bovine serum. The samples were incubated at 37°C for 6 h, followed by removal of free viruses with a wash in serum-free CO_2_-independent medium. Because of a difference in temperature requirement, the fluorescent dye, CCF2-AM, was then loaded into these cells by passive diffusion for 2 h at room temperature, following the β-lactamase loading kit protocol (Invitrogen, Carlsbad, CA). Following washing with serum-free CO_2_-independent medium to remove any residual extracellular dye, the cells were resuspended in CO_2_-independent medium supplemented with 10% fetal bovine serum and 2.5 mM probenecid. Subsequent to incubation at room temperature in the dark for 16 h, the cells were fixed with 4% paraformaldehyde at 4°C for 20 min. The cells were then subjected to flow cytometry analysis in a Beckman Dickinson LSRII cytometer.

### Env incorporation into virions

293T cells (1 × 10^6^) were transfected with proviral vectors. Viral supernatants were harvested and clarified 72 h post transfection and were pelleted through a 25% sucrose cushion by ultracentrifugation at 100, 000 × g for 2.5 h. The layers of supernatant and sucrose were carefully removed, and the resulting viral pellets were resuspended in 200 μl PBS (pH 7.2). The viral pellets were subjected to p24 ELISA, gp120 ELISA [59,82], and gp41 western blot (using mAb Chessie 8 as the primary Ab) to determine the amount of p24, gp120, and gp41. Incorporation was determined by calculating the ratio of gp120 and gp41 to p24.

## Competing interests

The authors declare that they have no competing interests.

## Authors' contributions

Sushma Bhakta, Liang Shang, and Eric Hunter participated in the design of the study and drafted the manuscript. Sushma J. Bhakta, Liang Shang, Daniel Claiborne and Jessica Prince carried out the experiments described in the manuscript. All authors read and approved the final manuscript.
